# Plasma apolipoprotein concentrations and occurrence of cardiovascular events in the general population: an exploratory analysis

**DOI:** 10.1016/j.athplu.2025.04.003

**Published:** 2025-05-08

**Authors:** Avedis Torossian, Annelise Genoux, Zichun Cai, Nathan Jolivet, Mikaël Croyal, Arsênio Rodrigues Oliveira, Sébastien Dejean, Nathalie Viguerie, Cendrine Cabou, Bertrand Perret, Jean Ferrières, Vanina Bongard, Laurent O. Martinez

**Affiliations:** a Faculté de Santé, Université Toulouse 3, Toulouse, France; b Institut des Maladies Métaboliques et Cardiovasculaires, I2MC, UMR1297, Inserm, Université de Toulouse, Université Toulouse III – Paul Sabatier (UPS), Toulouse, France; c IHU HealthAge, Toulouse, France; d Nantes Université, CNRS, INSERM, l'institut du Thorax, 44000, Nantes, France; e Nantes Université, CHU Nantes, CNRS, INSERM, BioCore, US16, SFR Bonamy, F-44000, Nantes, France; f CRNH-Ouest Mass Spectrometry Core Facility, 44000, Nantes, France; g Institut de Mathématiques de Toulouse, UMR5219, CNRS, Université de Toulouse, UT3, 31062, Toulouse, France; h Centre d’Epidémiologie et de Recherche en santé des POPulations (CERPOP), UMR 1295, Inserm, Université Toulouse, Université Toulouse III – Paul Sabatier (UPS), Toulouse, France

**Keywords:** Apolipoprotein, Lipoprotein, Cardiovascular events, Biomarkers, Cardiovascular risk

## Abstract

**Background and aims:**

The role of many apolipoproteins in cardiovascular disease (CVD) pathophysiology and their predictive potential remains unclear. This study examined the association between plasma concentrations of a broad panel of apolipoproteins and the occurrence of cardiovascular events in a general population.

**Methods:**

A nested case-control study was conducted within a cohort from Southwestern France. Baseline concentrations of apolipoproteins A-I, A-II, A-IV, B100, C-I, C-II, C-III, D, E, H, J, L1 and M were analyzed in 65 cases who experienced a cardiovascular event during the follow-up period, and in 65 controls matched for age and sex (mean age 60.9 ± 10.7 years; 66.9 % men; median follow up 9.3 years for controls, 6.2 years for cases). Baseline correlations were assessed using Spearman's coefficients.

Logistic regression and partial least squares discriminant analysis (PLS-DA) were used to evaluate associations with the occurrence of cardiovascular events.

**Results:**

Concentrations of apolipoproteins A-I, A-IV, C-I, D, H, J and M differed significantly between cases and controls. All expect apoM remained independently associated with cardiovascular events after adjustment for known risk factors. Additionally, PLS-DA revealed that the entire apolipoprotein panel explained 64 % of variance in case-control status with 60 % predictive accuracy, with apolipoproteins D, J, A-IV, H, and C-I contributing the most to group discrimination.

**Conclusions:**

This study identifies a novel panel of apolipoproteins (A-I, A-IV, C-I, D, H, and J) whose levels are associated with occurrence of cardiovascular diseases, independently of traditional risk factors. Further research is needed to confirm these findings and explore underlying mechanisms.

## Highlights

• In the general population, specific apolipoproteins (A-I, A-IV, C-I, D, H, J, M) are associated with cardiovascular events.
• Associations remain after adjusting for traditional cardiovascular risk factors.
• Apolipoprotein profiling enhances prediction of cardiovascular outcomes.
• Apolipoproteins are crucial indicators beyond traditional cardiovascular risk factors.

## Graphical abstract

**Figure:**
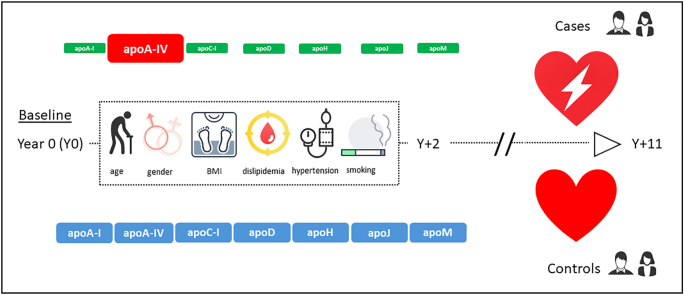

## 1 Introduction

Cardiovascular diseases represent a major public health issue worldwide [1,2], with events such as acute coronary syndrome and stroke accounting for the highest proportion of global mortality and significantly contributing to disability prevalence [[3], [4], [5]]. Apolipoproteins have become critical in cardiovascular research due to their central role in lipid metabolism and vascular functions [[6], [7], [8]]. Among these, major apolipoproteins are well-documented for their association with cardiovascular risk, with some showing greater predictive capacity for cardiovascular events compared to traditional plasma lipids, independently of conventional risk factors. For instance, plasma levels of apolipoprotein (apo) B100, the primary apolipoprotein of low-density lipoprotein (LDL), are positively associated with cardiovascular risk [[9], [10], [11], [12]], while apoA-I, the main apolipoprotein of high-density lipoprotein (HDL), is known for its protective, inverse association with cardiovascular risk [13]. However, the potential of other apolipoproteins—such as apoC-I, apoC-II, apoD, apoH, apoJ, apoL1, and apoM—as predictive marker of cardiovascular outcome remains largely underexplored, as their metabolic functions are less well understood [[14], [15], [16], [17]].

In this context, we conducted a nested case-control study within a cohort drawn from the general population of Southwestern France to examine the association between baseline plasma levels of 13 apolipoproteins (apoA-I, apoA-II, apoA-IV, apoB100, apoC-I, apoC-II, apoC-III, apoD, apoE, apoH, apoJ, and apoL1, apoM) and the occurrence of cardiovascular events.

## 2 Patients and methods

### 2.1 Study participants, follow-up and case ascertainment

Participants were drawn from the MONA LISA (MOnitoring NAtionaL du rISque Artériel) survey, whose protocol has already been extensively described elsewhere [18,19]. Briefly, between 2005 and 2007, women and men aged 35–74 years were randomly selected from electoral rolls in three French regions: Lille urban community in Northern France, Bas-Rhin department in Eastern France, and Haute-Garonne department in Southwestern France. The study protocol was approved by the local independent ethics committee in accordance with the French law. A collection of biological samples, registered as DC-2008-463 to the Ministry of Research and to the regional Health authority, was established and stored at the Toulouse-Bioressource biobank (TBR). Informed consent to participate was signed by all participants and all of the reported investigations were carried out in accordance with the principles of the Declaration of Helsinki.

**Fig. 1:**
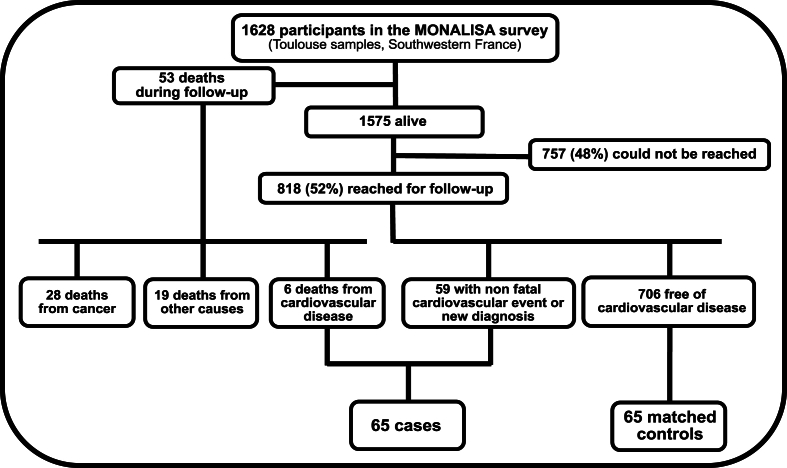
**Study flowchart**. Cases were individuals who developed fatal or nonfatal cardiovascular disease during follow-up: cardiovascular death, acute coronary syndrome (ST- or non-ST-elevation), coronary artery revascularization, stroke, transient ischemic attack, or diagnosis of significant peripheral artery stenosis or aorta aneurysm. Controls were individuals free of cardiovascular disease at baseline and during follow-up, matched with cases for age and sex. Median follow-up lengthened 9.3 years among controls (minimum 8.7; maximum 10.6) and 6.2 years among cases (min 0.6; max 9.6).

In 2016, a follow-up study was initiated for the 1628 participants recruited from Southwestern France. Vital status, along with the cause and date of death (if applicable), was determined using the French national database for death (CepiDC, https://www.cepidc.inserm.fr/). A total of 53 deaths were identified: 28 (53 %) from cancer, 6 (11 %) from cardiovascular disease and 19 (36 %) from other causes. Survivors were contacted by phone to ascertain the occurrence of cardiovascular events since their initial inclusion in 2005–2007. Of the 1575 individuals still alive, 818 (52 %) were successfully reached to collect follow-up information (Fig. 1). For participants who reported potential cardiovascular events, clinical data were collected from hospital medical records and/or general practitioners. Comprehensive information was collected on hospital admissions and types of medical interventions. Cardiovascular events considered in the study included cardiovascular deaths, acute coronary syndromes (both ST- and non-ST-elevation myocardial infractions), coronary artery revascularizations, strokes, transient ischemic attacks, and diagnoses of significant peripheral artery stenosis or aortic aneurysm.

### 2.2 Nested case-control study

Among participants with follow-up data, 65 individuals experienced cardiovascular events during the follow-up period. Of these, 8 had a history of cardiovascular disease at recruitment, while the remaining were newly diagnosed during follow-up. These participants formed the case group and were matched first for age and then for sex (men, women) with 65 participants who had no history of cardiovascular event at baseline and remained event-free during follow-up.

Analyses of baseline plasma apolipoprotein concentrations were conducted within this nested case–control population, consisting of 65 cases and 65 controls from the MONA LISA survey. The median follow-up duration was 9.3 years for controls (range: 8.7–10.6 years) and 6.2 years for cases (range: 0.6–9.6). The study workflow is illustrated and detailed in Fig. 1.

### 2.3 Baseline data collection (2005–2007)

At baseline, participants completed a standardized questionnaire on demographics, socioeconomic status, medical history, and medication use, and underwent a standardized physical examination. Body mass index (BMI) was calculated as weight (kg) divided by squared height (m^2^). Blood pressure was measured twice at rest using a standard sphygmomanometer (OMRON® 705IT). A fasting blood sample (20 mL) was collected after a minimum 10-h fast into a disodium ethylene-diamine-tetra acetic acid (EDTA) tube, and plasma was separated by centrifugation within 4 h.

Baseline standard biological measurements were performed in core laboratories: the Institut Pasteur de Lille and MShark mass spectrometry platform in Nantes. Plasma cholesterol and triglyceride concentrations were measured using enzymatic assays (Olympus, Melville, NY, USA). High-density lipoprotein cholesterol (HDL-C) was measured after sodium phosphotungstate/magnesium chloride precipitation (Olympus), and low-density lipoprotein cholesterol (LDL-C) was calculated using the Friedewald equation (for triglyceride concentrations <4.6 mmol/L) [20]. Fasting blood glucose (FBG) was measured by the glucose hexokinase assay (DuPont Dimension, Brussels, Belgium) while insulin and high-sensitivity C-reactive protein (hs-CRP) were assayed with immunoassays on an automated analyzer (Cobas 8000, Roche Diagnostics, Meylan, France). Circulating apolipoprotein levels were measured using liquid chromatography tandem mass spectrometry (LC-MS/MS) system, as previously described [21].

Cardiovascular risk factors included smoking (current smoking or cessation within the past three years), hypertension (systolic blood pressure [SBP] ≥140 mmHg and/or diastolic blood pressure [SBP] ≥90 mmHg at rest, and/or antihypertensive treatment), diabetes (FBG ≥1.26 g/L and/or hypoglycaemic treatment) and dyslipidemia. Dyslipidemia encompassed hypercholesterolemia (LDL-C ≥1,6 g/L and triglyceride levels <1.5 g/L and/or use of lipid-lowering drugs), hypertriglyceridemia (triglyceride levels ≥1.5 g/L and LDL-C levels <1.6 g/L), mixed dyslipidemia (LDL-C ≥1.6 g/L and triglyceride levels ≥1.5 g/L), and/or use of lipid-lowering treatment. The estimated glomerular filtration rate (eGFR) was calculated using the Chronic Kidney Disease Epidemiology Collaboration (CDK-EPI) equation [22], and eGFR lower than 90 and 60 mL/min/1.73 m^2^ were respectively labelled as mild and moderate or severe kidney disease. Physical activity was categorized into four levels: none, mild (up to one 20-min session of intense activity weekly), moderate (20 min up to twice weekly), and high (20 min ≥ 3 times weekly).

### 2.4 Statistical analysis

Categorical variables were summarized as percentages, and continuous variables as means ± standard deviation (SD) or medians [interquartile range, IQR]. Chi-square tests were used to compare the distributions of categorical variables between cases and controls, while differences between cases and controls were tested using Student's t- or Wilcoxon rank-sum tests for continuous variables. Assumptions for using tests were systematically checked before application. Correlations between continuous variables were analyzed and summarized with correlation matrices displaying Spearman correlation coefficients (SCC). Multivariate logistic regression models were used to analyse the contribution of plasma apolipoproteins as explanatory variables for determining case-control status and thus predicting occurrence of cardiovascular events. Each potential explanatory variable was associated with an odds ratio and a 95 % confidence interval (95 % CI). To account for potential confounding bias, adjustments were made for variables selected *a priori*, based on established cardiovascular risk factors, including age, sex (men, women), BMI, hypertension, diabetes, dyslipidaemia, and smoking. Continuous explanatory variables that did not exhibit a log-linear association pattern with case-control status (such as apoD, apoH and apoJ concentrations), were dichotomized as “low” (below median) or “high” (above median) levels. Since cases and controls were matched for age and sex (men, women), a conditional regression analysis was also conducted, as sensitivity analysis.

A supervised analysis was conducted using partial least squares discriminant analysis (PLS-DA) with cross validation to evaluate the ability of the multivariate model to discriminate between case and control individuals. Apolipoprotein concentrations were entered as continuous independent variables, and case-control status as dependent variable [23]. The Variable Importance Projection (VIP) score was used to identify the variables most contributive to the predictive ability of the PLS-DA model. PLS-DA analyses were performed with R version R-4.3.2 (package ropls). STATA® software version 18 (STATA Corporation, College Station, TX, USA) was used for all other analyses.

## 3 Results

### 3.1 Baseline characteristics of study population

The baseline clinical and biological characteristics of the study population are summarized in Table 1. It includes data for the entire cohort and the two groups categorized by case-control status based on cardiovascular events during the follow-up period.

**Table 1:** Baseline clinical and biological characteristics of the study population.

Characteristics	Whole population (n = 130)	Controls (n = 65)	Cases (n = 65)	p-value
Age, years	60.9 ± 10.7	61.1 ± 10.7	60.8 ± 10.9	0.885
Sex				
Men, n (%)	87 (66.9)	43 (66.1)	44 (67.7)	0.852
Women, n (%)	43 (33.1)	22 (33.8)	21 (32.3)	
BMI, kg/m^2^	27.1 ± 4.6	26.8 ± 4.6	27.3 ± 4.6	0.435
Smoking, n (%)				
Never smoked	63 (48.1)	31 (47.7)	32 (49.2)	0.649
Used to smoke (weaning >3 years)	50 (38.2)	27 (41.5)	23 (35.38)	
Currently smoking	17 (12.9)	7 (10.8)	10 (15.4)	
Total cholesterol, g/L	2.23 [1.98; 2.52]	2.30 [2.00; 2.65]	2.24 [1.96; 2.48]	0.435
LDL-C, g/L	1.49 ± 0.04	1.49 ± 0.06	1.49 ± 0.05	0.947
HDL-C, g/L	0.54 ± 0.01	0.57 ± 0.02	0.51 ± 0.01	0.006^a^
Triglycerides, g/L	1.05 [0.79; 1.49]	1.03 [0.78; 1.48]	1.07 [0.81; 1.53]	0.590
Fasting blood glucose, g/L	0.99 [0.92; 1.09]	1.00 [0.94; 1.06]	0.98 [0.89; 1.11]	0.721
Insulin, mU/L	4.6 [3.2; 7.1]	4.4 [2.9; 6.8]	4.7 [3.5; 7.7]	0.320
Dyslipidemia, n (%)	82 (62.6)	40 (61.5)	42 (64.6)	0.716
Hypercholesterolemia	33 (25.4)	16 (24.6)	17 (26.1)	0.840
Hypertriglyceridemia	17 (13.1)	9 (13.8)	8 (12.3)	0.795
Mixed dyslipidemia	15 (11.5)	6 (9.2)	9 (13.8)	0.410
Diabetes, n (%)	13 (10.0)	4 (6.1)	9 (13.8)	0.144
Hypertension, n (%)	83 (63.8 %)	39 (60.0)	44 (67.7)	0.361
Kidney function, n (%)				
Normal	19 (14.6)	5 (7.7)	14 (21.5)	
Mild kidney disease	90 (69.2)	48 (73.8)	42 (64.6)	0.118
Moderate or severe kidney disease	21 (16.1)	12 (18.46)	9 (13. %)	
hs-CRP, mg/L	1.31 [0.74; 2.98]	1.13 [0.55; 2.50]	1.52 [1.00; 3.40]	0.114^a^
Physical activity, n (%)				
None	47 (35.9)	24 (36.9)	23 (35.4)	
Mild	27 (20.6)	13 (20.0)	14 (21.5)	0.874
Moderate	40 (30.5)	21 (32.3)	19 (29.2)	
Intensive	15 (11.5)	7 (10.7)	8 (12.3)	

Categorical parameters are expressed as number and percentage. Continuous parameters are summarized as mean ± standard deviation (SD) or as median [first quartile, Q1; third quartile, Q3]. Between-group differences were tested using Chi-square test for categorical variables and Student's t-test for continuous variables. The Wilcoxon rank-sum test was used when distribution of continuous variables significantly departed from normality. Diabetes was defined as fasting blood glucose ≥ ≥1.26 g/L and/or hypoglycaemic treatment. Dyslipidemia encompassed hypercholesterolemia (LDL-C ≥1,6 g/L and triglyceride levels <1.5 g/L and/or use of lipid-lowering drugs), hypertriglyceridemia (triglyceride levels ≥1.5 g/L and LDL-C levels <1.6 g/L), mixed dyslipidaemia (LDL-C ≥1.6 g/L and triglyceride levels ≥1.5 g/L), and/or use of lipid-lowering treatment. Hypertension was defined as systolic blood pressure ≥140 mmHg or diastolic blood pressure ≥90 mmHg or treatment. Physical activity was categorized into four levels: none, mild (up to one 20-min session of intense activity weekly), moderate (20 min up to twice weekly), and high (20 min ≥ 3 times weekly).

BMI, body mass index; HDL-C, High density Lipoprotein cholesterol; hs-CRP, High-sensitivity C-Reactive Protein; LDL-C, Low density Lipoprotein cholesterol.

a Wilcoxon rank-sum test.

The two matching parameters used in the nested case-control study design —age and sex— were well balanced between cases and controls. The mean age was 60.9 years, and 66.9 % of participants were men. Apart from HDL-C, which was significantly lower among cases (0.50 g/L) compared to controls (0.56 g/L, p = 0.005), no significant difference was observed between the two groups.

The mean BMI was 27.1 ± 4.6 kg/m^2^, indicating overweight status. Approximately 63 % of participants had high blood pressure, and 63 % dyslipidaemia, with 25.4 % experiencing hypercholesterolemia, 13 % hypertriglyceridemia, and 11.5 % mixed dyslipidaemia. Additionally, 10 % of participants had FBG levels exceeding 1.26 g/L, or were undergoing diabetes treatment.

Triglycerides and LDL-C did not differ between cases and controls. Regarding non-lipid biochemical parameters, most participants had reduced eGFR, consistent with mild kidney disease, which was expected given the cohort's average age. Although hs-CRP levels were higher among cases, the difference with control participants was not statistically significant.

Finally, over 63 % of participants declared having regular physical activity at mild to high levels, and 48.5 % reported never smoking (Table 1).

### 3.2 Baseline plasma concentrations of apolipoproteins and major cardiovascular events during follow-up

The concentrations of apoA-I, apoC-I, apoD, apoH, apoJ and apoM were significantly lower in the case, compared to the control group (Table 2). Among these apolipoproteins, apoA-I and apoM showed the smallest differences, with concentrations 8 % and 10 % lower in the case group, respectively. In contrast, apoD and apoJ showed the largest differences, with their levels approximately 38 % lower in the case group. Levels of apoH and apoC-I were 16 % and 20 % lower, respectively. Conversely, among all the apolipoproteins analyzed, only apoA-IV showed an increase in concentration in the case group, with levels 28 % higher than in the control group. For the remaining apolipoproteins, including apoA-II, apoB100, apoC-II, apoC-III, apoE and apoL1, there were no significant differences in plasma concentrations between the case and control groups (Table 2).

**Table 2:** Baseline plasma concentration of apolipoproteins according to case-control status.

Apolipoproteins	Controls (n = 65)	Cases (n = 65)	p-value
**ApoA-I**, mg/dL	**128 ± 27**	**118 ± 22**	**0.022**^a^
ApoA-II, mg/dL	25.4 ± 5.1	25.6 ± 4.4	0.757^a^
**ApoA-IV**, mg/dL	**6.29 [1.84]**	**8.07 [3.66]**	**<0.0001**^b^
ApoB100, mg/dL	91 ± 19	89 ± 24	0.785^a^
**ApoC-I**, mg/dL	**2.66 ± 0.83**	**2.12 ± 0.77**	**<0.0001**^a^
ApoC-II, mg/dL	2.30 ± 0.99	2.36 ± 0.97	0.597^a^
ApoC-III, mg/dL	6.43 [2.19]	6.03 [1.72]	0.336^b^
**ApoD**, mg/dL	**10.16 ± 2.61**	**6.28 ± 1.88**	**<0.0001**^a^
ApoE, mg/dL	3.31 ± 1.14	3.27 ± 1.21	0.827^a^
**ApoH**, mg/dL	**5.60 ± 1.24**	**4.72 ± 1.18**	**<0.0001**^a^
**ApoJ**, mg/dL	**14.65 ± 4.02**	**9.06 ± 3.68**	**<0.0001**^a^
ApoL1, mg/dL	1.56 ± 0.43	1.51 ± 0.36	0.501^a^
**ApoM**, mg/dL	**2.44 [0.94]**	**2.19 [0.58]**	**0.049**^b^

Apolipoprotein concentrations are expressed as the mean ± SD in the case of Gaussian distribution, otherwise as the median [interquartile range, IQR].

a Student's T-test.

b Wilcoxon rank-sum test.

### 3.3 Correlations between baseline plasma apolipoprotein levels and cardiovascular risk parameters

**Fig. 2:**
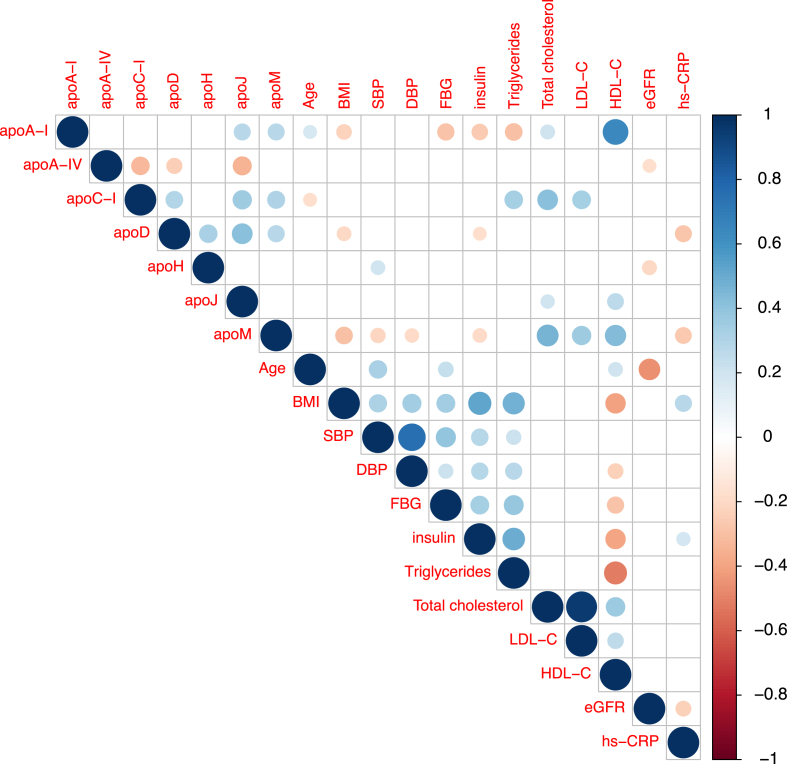
**Spearman correlations between plasma apolipoprotein concentrations and bio-clinical variables in the whole study population**. Plasma apolipoprotein concentrations were measured in the whole study population (n = 130). Size and color, according to the color scale detailed on the right, represent the strength of the correlation intensity between variables, with Spearman's r ranging from -1 to +1. Only correlations significant at the 5 % threshold are shown. Non-significant correlations are indicated by empty boxes. Blue dot, positive correlation; red dot, negative correlation. BMI, Body mass index; DBP, diastolic blood pressure; eGFR, estimated glomerular filtration rate; FBG, fasting blood glucose; hs-CRP, high-sensitivity C-reactive protein; SBP, systolic blood pressure (SBP).

Correlations were assessed between plasma concentrations of apolipoproteins that exhibited significant differences between case and control groups and cardiovascular risk parameters (Fig. 2). Levels of apoA-I, apoC-I, and apoD were positively correlated with those of apoJ and apoM, with additional positive correlations observed between apoC-I and apoD, as well as between apoD and apoH. In contrast, apoA-IV levels were negatively correlated with apoC-I, apoD and apoJ. This aligns with the finding that apoA-IV was the only apolipoprotein elevated in the case group, while apoA-I, apoC-I, apoD, apoH, apoJ, and apoM levels were significantly reduced in this group (Table 2).

Notable associations between apolipoprotein levels and cardiovascular risk factors include negative correlations of apoA-I, apoD, and apoM with BMI and insulin levels. Additionally, apoM and apoD were negatively associated with hs-CRP, with apoM also showing negative correlation with systolic and diastolic blood pressure.

Regarding plasma lipids, apoA-I, apoJ and apoM were positively correlated with HDL-C. Additionally, apoA-I showed a negative correlation with triglycerides, while apoM was positively associated with total cholesterol and LDL-C. ApoC-I was positively correlated with triglycerides, total cholesterol, and LDL-C.

### 3.4 Baseline plasma apolipoprotein concentrations and risk of cardiovascular events

We first performed unadjusted logistic regression analyses, using case-control status as the dependent variable and each apolipoprotein previously identified as differentially expressed between cases and controls as an independent continuous variable (Table 3). All analyzed apolipoproteins—specifically apoA-I, apoA-IV, apoC-I, apoD, apoH, apoJ and apoM—showed a significant association with case-control status. For apolipoproteins D, H and J, whose expression levels did not exhibit a log-linear relationship with case-control status, we conducted further analyses by categorizing their expression levels as high or low, corresponding to values above or below their respective median levels (Table 3). These additional analyses confirmed significant associations between these apolipoproteins and case-control status. Notably, with the exception of apoA-IV (the only apolipoprotein whose plasma levels were higher in the case group), all odds ratios were significantly below 1, indicating that higher levels of these apolipoproteins were associated with a reduced probability of cardiovascular events during follow-up. Conversely, higher levels of apoA-IV were associated with increased probability of such events.

**Table 3:** Logistic regression models assessing case-control status with plasma apolipoprotein levels as independent variables.

	Unadjusted			Model 1			Model 2		
	OR	95 % CI	p-value	OR	95 % CI	p-value	OR	95 % CI	p-value
*Per 1-unit increase*									
**apoA-I**	**0.98**	0.96–0.99	0.026	**0.98**	0.96–0.99	0.040	**0.97**	0.95–0.99	0.022
**apoA-IV**	**1.53**	1.25–1.87	<0.001	**1.56**	1.25–1.94	<0.001	**1.54**	1.23–1.95	<0.001
**apoC-I**	**0.36**	0.20–0.64	0.001	**0.34**	0.18–0.63	0.001	**0.29**	0.15–0.58	<0.001
**apoD**	**0.45**	0.34–0.58	<0.001	**0.38**	0.28–0.53	<0.001	**0.35**	0.24–0.50	<0.001
**apoH**	**0.54**	0.39–0.74	<0.001	**0.52**	0.37–0.73	<0.001	**0.47**	0.30–0.68	<0.001
**apoJ**	**0.67**	0.59–0.77	<0.001	**0.64**	0.55–0.75	<0.001	**0.56**	0.46–0.69	<0.001
**apoM**	**0.43**	0.22–0.84	0.014	0.50	0.25–1.03	0.062	0.48	0.21–1.21	0.091

*High versus Low level*									
**apoD**	**0.06**	0.02–0.14	<0.001	**0.04**	0.01–0.11	<0.001	**0.02**	0.01–0.09	<0.001
**apoH**	**0.29**	0.14–0.61	0.001	**0.33**	0.15–0.69	0.003	**0.26**	0.11–0.59	0.002
**apoJ**	**0.09**	0.03–0.20	<0.001	**0.07**	0.02–0.18	<0.001	**0.02**	0.01–0.09	<0.001

Model 1: Adjusted for age, sex and BMI.

Model 2: Model 1 + dyslipidemia, hypertension, diabetes and smoking.

High and low levels correspond to value above and below the median plasma apolipoprotein levels: 7.76 mg/dL, 5.18 mg/dL, and 12.05 mg/dL for apoD, apoH and apoJ, respectively.

The standard error (SE), 95 % confidence interval (95 % CI), and p-value associated with each odds ratio (OR) are reported.

Dyslipidemia was defined as LDL-C ≥ 1,6 g/L and/or triglyceride levels <1.5 g/L and/or lipid-lowering treatment. Hypertension was defined as systolic blood pressure (SBP) ≥ 140 mmHg and/or diastolic blood pressure (DBP) ≥ 90 mmHg at rest, and/or antihypertensive treatment. Diabetes was defined as fasting blood glucose (FBG) ≥ 1.26 g/L and/or hypoglycaemic treatment.

BMI, body mass index.

To address potential confounding factors, we conducted two additional analyses, adjusting the initial model for variables commonly associated with cardiovascular events: (1) age, sex and BMI (model 1), and (2) age, sex, BMI, dyslipidemia, hypertension, diabetes and smoking (model 2). With the exception of apoM levels, which displayed a p-value marginally above the significance threshold in models 1 and 2 (p = 0.062 and p = 0.091, respectively), all other apolipoprotein levels remained significantly associated with case-control status. Further adjustment for HDL-C did not result in any notable change (not shown). Furthermore, since cases and controls were matched, we conducted a conditional regression analysis, which yielded results consistent with the conventional logistic approach (not shown).

**Fig. 3:**
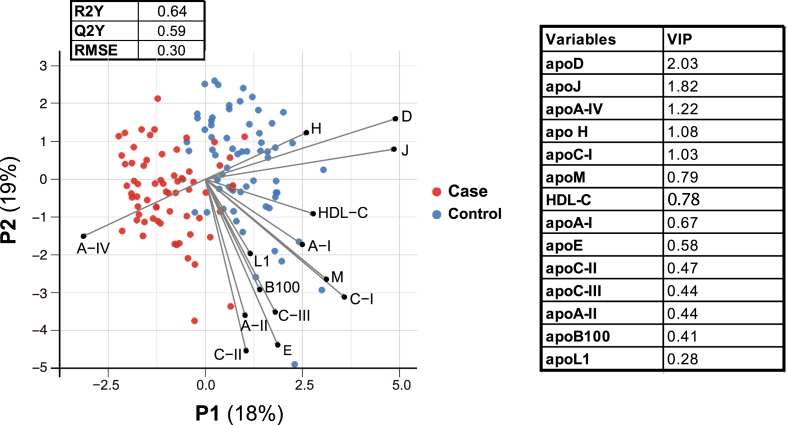
**Relationship between plasma apolipoprotein and HDL-C concentrations and case-control status** PLS-DA analysis was used to explain case-control status based on the set of plasma apolipoprotein concentrations, along with HDL-C levels, in the whole study population (n = 130 individuals). The plot displays PLS-DA score for each individual and the grey straight lines represent loading coefficients for each apolipoprotein variables correlated with the first two components, P1 and P2. Predictive performance (Q2Y), goodness of model fit (R2Y), and RMSE values are provided (upper left table), along with the VIP values (right table). Blue dot, control; red dot, case. PLS-DA, partial least squares discriminant analysis; RMSE, root mean square error; VIP, variable importance projection.

To further evaluate the ability of plasma apolipoprotein concentrations to differentiate cases from controls, we conducted a PLS-DA analysis of case-control status based on the entire panel of plasma apolipoproteins, including HDL-C, whose levels were lower in cases (Table 1), while PLS regression addressed multicollinearity among the variables (Fig. 3). The first two components, P1 and P2, accounted for 37 % of the model variability. The PLS-DA model showed a strong predictive capability for distinguishing cases from controls, with R2Y = 0.64 and Q2Y = 0.60, where R2Y indicates the cumulative variation in case-control status explained by P1 and P2, and Q2Y represents the model's predictive accuracy based on cross-validation. The loading coefficients on the PLS-DA score plot revealed distinct clustering patterns: apoD, apoJ, and apoH showed opposite trends along the P1 component compared to apoA-IV, while a separate pattern emerged, including HDL-C, apoA-I, apoA-II, apoB100, apoC-I, apoC-III, apoE, and apoL1. Based on variable importance in projection (VIP) scores, concentrations of apoD, apoJ, apoA-IV, apoH, and apoC-I contributed the most to the model's predictive power (Fig. 3, right inset table).

## 4 Discussion

This study investigated the association between baseline plasma levels of a panel of 13 apolipoproteins and cardiovascular disease (CVD) risk in a population-based nested case-control cohort. The cohort included 65 case patients who experienced cardiovascular events during follow-up and 65 age- and sex-matched control participants who remained event-free.

PLS-DA analysis revealed that the apolipoprotein panel collectively explained 64 % of the variance in case-control status, with a predictive accuracy of 60 %. Among the apolipoproteins, apoD, apoJ, apoA-IV, apoH, and apoC-I showed the highest predictive power, with VIP scores >1. This finding highlights their potential as key biomarker for CVD risk prediction in the general population. Notably, these apolipoproteins demonstrated consistent associations with the case-control status across all regression models.

Most of the differentially expressed apolipoproteins—apoA-I, apoC-I, apoD, apoH, apoJ and apoM—were found at lower baseline levels in individuals who later experienced CVD, except for apoA-IV. ApoD and apoJ showed the largest differences (up to -40 %) in levels between cases and controls and were both negatively associated with CVD risk, independently of traditional risk factors, comorbidities and treatments.

ApoD, a member of the lipocalin superfamily, primarily binds arachidonic acid [24,25]. We observed that its plasma levels negatively associated with BMI, insulin and hs- CRP, supporting its role in limiting body fat accumulation, protecting against hyperinsulinemia and myocardial infarction [[26], [27], [28]] as well as its antioxidant and anti-inflammatory properties [29,30]. However, one study showed that high circulating levels of apoD are associated with poor prognosis in coronary artery disease [31]. Unlike our analysis, which accounted for potential confounders and treated apoD levels as a continuous variable, this study categorized apoD levels into quartiles and lacked data on key medications such as lipid-lowering drugs, antihypertensives, or antidiabetic agents—important confounders that we were able to adjust for. Additionally, the non-linear relationship between apoD (as well as apoJ and apoH) and case-control status might also explain this discrepancy. Nevertheless, our study confirmed a negative and independent association between apoD levels and CVD risk, even when plasma levels were categorized into high and low groups around the median value (Table 3), further supporting our conclusions.

ApoJ, also known as Clusterin, is a molecular chaperone with anti-oxidant and cytoprotective properties [32]. We found that its plasma levels positively correlate with HDL-C levels, consistent with its transport by HDL particles. This association aligns with described HDL-mediated atheroprotective role of apoJ, such as promoting HDL-mediated reverse cholesterol transport (10.13039/100014144RCT) from peripheral tissue to the liver for excretion, or supporting HDL-associated paraoxonase 1 activity, which protects against LDL oxidation [29,31,32]. Additionally, ApoJ might contribute to reducing CVD risk through its pleiotropic functions, including its regulation of glucose metabolism and its involvement in the endocrine Clusterin/LDL Receptor Related Protein 2 (LRP2) axis [33]. Glycosylation, a key post-translational modification of the ApoJ protein, influences its function, with lower levels of glycosylated ApoJ (ApoJ-Glyc) observed during acute ischemic events, suggesting its potential as a myocardial ischemia biomarker [34,35]. Our study, however, focuses on a different context—specifically, outside the acute phase—and analyzes total ApoJ levels rather than its glycosylated forms. It suggests that lower total ApoJ levels may serve as an upstream biomarker, potentially indicating cardiovascular risk well before the onset of clinical events.

ApoH exhibited lower baseline concentrations in cases, contrasting with previous reports linking higher apoH levels to an increased risk of CVD [36]. This discrepancy may arise from the varying contributions of apoH isoforms to CVD risk, highlighting a complex relationship [37]. Additionally, the observed non-linear association between apoH levels and case-control status suggests a potential threshold-dependent effect, warranting further investigation into the underlying mechanisms.

Conversely, apoC-I exhibited a linear relationship with cardiovascular outcomes, indicating a dose-dependent mechanism underlying this association. ApoC-I is known to be involved in both very low-density lipoprotein (VLDL) and HDL metabolism with conflicting roles regarding cardiometabolic risk—most notably through its role in inhibiting cholesteryl ester transfer protein (CETP), depending on the overall metabolic context [38,39]. In our study, plasma concentrations of apoC-I did not correlate with HDL-C levels, suggesting that apoC-I may contribute to a HDL-mediated cardiovascular protective effect through regulatory mechanisms, rather than through a direct quantitative association. Furthermore, apoC-I may be linked to cardiovascular risk through HDL-independent mechanisms, given its regulatory roles across diverse aspects of cardiovascular physiology, including inflammation, immunity, sepsis, diabetes, cancer and viral infectivity [40].

ApoA-IV presented an intriguing finding: its levels were higher in cases, contrary to prior reports of an inverse association with coronary artery disease, primarily attributed to its role in inhibiting vascular inflammation by preventing NF-κB activation [41]. It has also been proposed that apoA-IV may protect against atherosclerosis by facilitating RCT [42]. It remains to be determined whether the higher levels of apoA-IV among case participants serve as a compensatory mechanism in response to impaired RCT, or alternatively reflect an unknown role in CVD progression, or are merely a passive bystander.

Although apoM and apoA-I exhibited smaller differences between groups, apoA-I retained significant associations with case-control status after adjustment for traditional risk factors, consistent with its known atheroprotective effects [13,16,43]. ApoM, like apoD, is a member of the lipocalin superfamily, and serves as a carrier of sphingosine 1-phosphate (S1P) within HDL particles [44]. In line with data from the literature, levels of apoM negatively correlated with metabolic and cardiac-related parameters, including BMI, fasting insulin, as well as hs-CRP [45]. However, its association with CVD occurrence appeared to depend on adjustments for traditional risk factors in logistic regression analyses.

To the best of our knowledge, we are the first to explore a large panel of circulating apolipoproteins to predict cardiovascular events, demonstrating predictive capacity independent of traditional risk factors. Nevertheless, this study has several limitations, the most being the small sample size. This limitation raises the possibility that non-significant trends observed at baseline between groups may reflect true differences that our statistical tests were underpowered to detect. Additionally, we were able to reach only 52 % of the participants in the MONA LISA survey for follow-up data collection, which might have introduced selection bias, although its impact remains difficult to assess. Another methodological limitation concerns data collection: information on CVD and case-control status was primarily self-reported and data were gathered by telephone, without oversight from an independent adjudication committee. This approach may have introduced classification bias. Finally, despite adjustment for a panel of potential confounders, including traditional CVD risk factors, residual confounding cannot be excluded. Consequently, our results should primarily be interpreted in an exploratory context, with the need for further validation in large-scale, population-based prospective cohort studies.

In conclusion, our study provides promising insights into the potential of certain lesser-known apolipoproteins as predictive markers of CVD risk, either individually or as part of a biomarker panel, independent of classical risk factors. If validated, it would be of particular interest to investigate whether specific apolipoproteins can predict distinct types of cardiovascular event and/or operate within specific timeframes. Overall, our study underscores the untapped clinical potential of apolipoproteins as novel indicators for cardiovascular risk assessment and management.

## CRediT authorship contribution statement

**Avedis Torossian:** Methodology, Data curation, Formal analysis, Writing – original draft. **Annelise Genoux:** Methodology, Funding acquisition, Supervision. **Zichun Cai:** Methodology, Data curation, Formal analysis. **Nathan Jolivet:** Methodology, Data curation, Formal analysis. **Mikaël Croyal:** Methodology, Validation, Supervision, Writing – review & editing. **Arsênio Rodrigues Oliveira:** Methodology, Formal analysis, Data curation. **Sébastien Dejean:** Methodology, Supervision, Writing – review & editing. **Nathalie Viguerie:** Methodology, Supervision, Writing – review & editing. **Cendrine Cabou:** Investigation, Writing – review. **Bertrand Perret:** Investigation, Writing – review. **Jean Ferrières:** Conceptualization, Investigation, Writing – review. **Vanina Bongard:** Conceptualization, Investigation, Supervision, Writing – review & editing. **Laurent O. Martinez:** Conceptualization, Funding acquisition, Investigation, Supervision, Writing – review & editing.

## Declaration of generative AI and AI-assisted technologies in the writing process

During the preparation of this work the authors did not use AI and AI-assisted Technologies.

## Fundings

The MONA LISA Study was made possible by an unrestricted grant from Pfizer and by a grant from the Agence Nationale de la Recherche ANR (ANR-05-PNRA-018). This specific work was supported by a grant from the Federation Française de Cardiologie (Dotation Recherche # FFC – MARTINEZ – Dotation 2022) and conducted in the context of IHU HealthAge, which has benefited from funding from the ANR under the France 2030 program (ANR-23-IAHU-0011).

## Declaration of competing interest

The authors declare that they have no known competing financial interests or personal relationships that could have appeared to influence the work reported in this paper.
